# Human CD133-positive hematopoietic progenitor cells enhance the malignancy of breast cancer cells

**DOI:** 10.1186/s12885-020-07633-3

**Published:** 2020-11-26

**Authors:** Zhe Zhang, Qinglian Zheng, Yonghui Liu, Lianqing Sun, Pingping Han, Rui Wang, Jiao Zhao, Shan Hu, Xinhan Zhao

**Affiliations:** 1 Department of Traditional Chinese Medicine, the First Affiliated Hospital of Medical School of Xi’an Jiaotong University, Xi’an, Shaanxi 710061 People’s Republic of China; 2 Department of Medical Oncology, the First Affiliated Hospital of Medical School of Xi’an Jiaotong University, Xi’an, Shaanxi 710061 People’s Republic of China

**Keywords:** CD133, Hematopoietic progenitor cells, Breast cancer cells, Malignancy

## Abstract

**Background:**

Human CD133+ hematopoietic progenitor cells (HPCs) are a specific subset of cells that can regulate tumor malignancy. However, the mechanism by which CD133+ HPCs affect the malignancy of human breast cancer has not been reported.

**Methods:**

CD133+ HPCs were isolated and purified from human umbilical cord blood (UCB). We used in vitro culture of MCF-7 and MDA-MB-231 cell lines, and MCF-7 and MDA-MB-231 cells in nude mice to evaluate whether CD133+ HPCs affected the apoptosis, proliferation, invasion and epithelial mesenchymal transition EMT of breast cancer cells.

**Results:**

Co-culture with CD133+ HPCs, but not UCB CD133- cells, promoted the proliferation of human breast cancer MCF-7 and MDA-MB-231 cells, accompanied by reducing in vitro spontaneous apoptosis. Co-administration of these two lines with CD133+ HPCs significantly enhanced the growth of implanted breast cancer in vivo. Furthermore, co-culture with CD133+ HPCs, enhanced the invasion of breast cancer cells, N-cadherin and Vimentin expression, but reduced E-cadherin expression in breast cancer cells.

**Conclusions:**

Our study demonstrated that CD133+ HPCs enhance the malignancy of breast cancer cells by attenuating spontaneous apoptosis and promoting the process of epithelial mesenchymal transition. These findings may provide new insights into the role of human CD133+ HPCs in breast cancer pathogenesis. Therefore, CD133+ HPCs may be a new therapeutic target for inhibiting the progression of breast cancer.

**Supplementary Information:**

The online version contains supplementary material available at 10.1186/s12885-020-07633-3.

## Background

Breast cancer metastasis is a significant cause of cancer-related death in women. Cancer metastasis is a dynamic process and is regulated by many factors. During cancer metastasis, malignant tumor cells are subjected to a series of changes, including alterations in cytoskeleton, morphology, proliferation and invasion capacities to enhance malignancy. It is notable that malignant tumor cells often undergo epithelial mesenchymal transition (EMT), characterized by loss of epithelial markers (e.g., E-cadherin) and acquisition of mesenchymal N-cadherin and Vimentin expression. However, it is unclear how these factors regulate the EMT process and metastasis in breast cancer [1, 2].

The development of metastases is influenced by an intricate interaction between breast cancer cells and the microenvironment [3]. Prior work has shown that cells existing in the metastatic microenvironment are recruited from bone marrow derived cells [4]. In particular, the bone marrow-derived hematopoietic progenitor cells (HPC) are involved in the initiation of metastases [5]. HPCs are primitive cells predominantly in hematopoietic tissues, such as the bone marrow and umbilical cord blood (UCB) [6–8]. HPCs can proliferate and differentiate into various kinds of blood cells. Human HPCs can be recognized by their surface CD133 and CD34 expression [9–13]. The early developing human CD133+ HPCs are a subpopulation of cells in the bone marrow, fetal liver, UCB and peripheral blood [14–18]. Functionally, CD133+ HPCs have stronger proliferation and migration potential than CD133- cells, perhaps, representing more primitive hematopoietic cells [19, 20]. A recent study suggested that CD133+ human umbilical hematopoietic progenitor cells may induce proliferation or metastasis of colorectal cancer cells [21]. However, few experiments have addressed the role of HPCs in breast cancer. Furthermore, clinical studies indicate that transplantation with hematopoietic stem cells after high dose of chemotherapies benefits patients with advanced breast cancer [22, 23]. However, there is little information on how human HPCs regulate the malignancy of breast cancer.

UCB can be obtained easily and is an excellent alternative source of HPCs. In this study, we isolated human CD133+ HPCs and CD133- cells from human UCB and tested whether CD133+ HPCs modulated the malignancy of breast cancer cells in vitro and in vivo to explore the potential mechanisms involved in metastasis.

## Methods

### Isolation of CD133+ HPCs

The Human Ethics Committee of the First Affiliated Hospital, College of Medicine at Xi’an Jiaotong University approved the experimental protocol (2017–041). Written informed consent was signed the parents of individual newborns. Fresh and healthy human UCB samples (about 60–100 ml) were collected from five human newborns in our hospital. The CD133+ HPCs were isolated by immunomagnetic beads and Magnetic Activated Cell Sorting (MACS) column (Mitenyi Biotec, Germany) according to the manufacturer’s instructions. Briefly, the mononuclear cells in the UCB samples were isolated by density gradient centrifugation at 2000 rpm for 25 mins using human lymphocyte separation solution. After being washed, the collected mononuclear cells (1 × 10^8^ cells/sample) in 5 μg/ml BSA (Merck, Germany) and 2 mmol/ml EDTA buffer (Promega, USA) were blocked with anti-CD16/anti-CD32 (Promega, USA) and stained with anti-human CD133 immunomagnetic beads, followed by loading into the Macs column. During column washing, the flow-through CD133- cells were collected and the bound CD133+ HPCs were eluted. The CD133+ and CD133- cells were stained with PE-anti-CD133 (MBS851590, MyBioSource, California, USA) and FITC-anti-CD34 (MBS850595, MyBioSource, California, USA). The percentages of CD133 + CD34+ cells were analyzed by flow cytometry, the remaining cells were designated CD133- human umbilical cord blood cells (HUCBCs). The isolated CD133 + CD34+ HPCs were routine-cultured in IMDM (Novagen, USA) medium containing 10% of fetal bovine serum (FBS, MyBioSource), and semi suspended in a 37 °C / 5% CO_2_ incubator. Cell growth was monitored daily.

### Culture of breast cancer cells

MCF-7 and MDA-MB-231 breast cancer cell lines were purchased from Shanghai cell bank of the Chinese Academy of Sciences. The cells were cultured with DMEM (MCLAB, USA) containing 10% newborn bovine serum (MCLAB, USA) under the conditions of 37 °C and 5% CO_2_. 0.25% trypsin (MCLAB, USA) was used to digest and passage. Fresh medium was replaced every 2–3 days.

### Cell proliferation assay

The effect of CD133+ HPCs on the proliferation of breast cancer cells was quantified by 3-(4,5-dimethylthiazol-2-yl)-2,5-diphenyltetrazolium bromide (MTT) assays [24]. MCF-7 and MDA-MB-231 breast cancer cells in logarithmic growth period were inoculated in 96 well plates, 2 × 10^3^ breast cancer cells for each group. After 12 h of culture, CD133+ HPCs and CD133- HPCs were added to the experimental group and the negative control group in a 20:1 proportion of breast cancer cells to HPCs. 24 h later, each well was dosed with 200 μL serum-free medium DMEM and 20 μl 5 mg/ml MTT (MCLAB, USA). After 4 h, 150 μL of DMSO (MCLAB, USA) was added to each well and incubated at room temperature for 10 min, oscillated with a micro-oscillator for 15 min, and optical density value was recorded at 570 nm with a flow cytometer instrument. All measurements were performed in triplicate.

### Transwell invasion assay

We examined the influence of CD133+ HPCs on breast cancer cell invasion by Transwell invasion assay [25]. Briefly, the upper and lower chambers of a Transwell insert are separated by polycarbonate microporous membrane (8 μm pore size), which is coated with Matrigel (Thermo Scientific, USA) on the upper chamber surface and dried at room temperature. 1 × 10^5^ breast cancer cells were added to the upper chamber. CD133+ HPCs and CD133- HPCs were added to the lower chamber of the experimental group and the negative control group. The cell number ratio of breast cancer cells to HPCs was 20:1. After culturing at 37 °C for 24 h, the non-invasive cells were wiped off with a cotton swab. The filter membrane was fixed with 4% paraformaldehyde solution (Thermo Scientific, USA) for 30 min, and 0.01% crystal violet dye solution was added for a 20 min incubation. The migrating cells were photoimaged and counted under the microscope. All measurements were performed in triplicate, and the experiments were repeated three times independently.

### Cell apoptosis assay

We tested the role of CD133+ HPCs in spontaneous apoptosis of MCF-7 or MDA-MB-231 cells by flow cytometry [26]. The cancer cells were cultured alone (blank control), or together with CD133+ HPCs or CD133- HUCBCs at a proportion of 20:1 in the upper and lower chambers of the Transwell insert (0.4 μm pore size), respectively, for 72 h. The cancer cells (3 × 10^5^ cells/tube) were tested for apoptosis by flow cytometry after staining with 5 μL Annexin V-FITC (Promega, USA) and 10 μL Propidium iodide (PI, MyBioSource). The apoptotic FITC+ and FITC+PI+ cells were quantified and the experiment was repeated three times.

### Western blotting

The Transwell (0.4 μm) indirect co-culture method and groups were described as above. The MCF-7 and MDA-MB-231 breast cancer cells were digested with 0.25% trypsin, and 250 μL RIPA lysate was added, incubated on ice for 10 min, and centrifuged at 4 °C for 20 min. Following centrifugation, the supernatant was collected and the protein concentration of each group of samples was detected on the spectrophotometer. A 10% separation gel and 5% concentrated gel were prepared, a 20 μg protein was mixed with 2 × SDS sample buffer in a 1:1 ratio. Samples were added to the gel followed by electrophoresis (concentrating gel 80 V; separating gel 100 V). The gel interlayer was removed and the target protein and β-actin were isolated according to molecular weight. The proteins were transferred to a PVDF membrane followed by soaking the membrane in methanol. The membrane was placed in 5% skimmed milk powder prepared by TBST for non-specific antigen blocking, and then Rabbit anti rat E-cadherin antibody (Merck, Germany, diluted in 1:2000), Rabbit anti rat Vimentin (Merck, Germany, diluted in1:500), Rabbit anti rat N-cadherin antibody (Merck, Germany, diluted in 1:500), or Rabbit anti rat β-actin antibodies were added (Merck, Germany, diluted in 1:2000). The membrane was washed three times with TBST, then Sheep anti rabbit IgG (Merck, Germany, diluted in 1:5000) labeled by HRP (Merck, Germany) was added for incubation followed by 3x TBST wash. After the film dried slightly, it was incubated with supersignal chemiluminescent reagent (Merck, Germany). The membrane was placed in a dark box and exposed together with the X-ray film (Merck, Germany). The exposure time was about 1 min. X-light was photographed after developing and fixing, and the gray value of the strip was analyzed by gel image analysis software IMAGE-J.All measurements were performed in triplicate, and the experiments were repeated three times independently.

### Animal experiment

Six-week-old female BALB/c nude mice were obtained from Silaike Laboratory Animal Co., Ltd.,Shanghai, China. The protocol was authorized by the Animal Care and Use Committee of Xi’an Jiaotong University. In order to evaluate whether CD133+ HPCs affected the growth of breast cancer in vivo, a 1 × 10^7^ suspension cells of MCF-7 and MDA-MB-231 breast cancer cells were taken respectively, mixed with CD133+/− cells, and inoculated to the nude mice (*n* = 8 in each group). In the experimental and negative control group, breast cancer cells and CD133+/− cells were added in a 20:1 ratio. For the negative control group, breast cancer cells and CD133- HPCs cell suspensions were added in a 20:1 ratio. For the blank control group, only breast cancer cells were inoculated. Tumor growth was monitored every 2 days for 30 days after inoculation, and the tumor volume (volume = length×width2 × 0.5) was measured. Each group of eight mice were housed in separated individual standard cleaned cages under automatically controlled air conditioning system with temperature (22 ± 2 °C), humidity (about 60%), and lighting (12:12-h light–dark cycle). Diet and sterilized water are provided in the experiments. At the endpoint, mice were euthanized by inhalation of CO_2_ followed by cervical dislocation. On the 30th day, mice were sacrificed and the tumor tissues were collected, stained with hematoxylin and eosin (H&E) and observed under an optical microscope.

### Statistical analysis

Data are expressed as mean ± SD. We statistically analyzed the significance among groups by one way ANOVA and between groups by Student’s *t* test using the SPSS 22.0 software. A *P*-value of < 0.05 was defined as statistical significance.

## Results

### Isolation and characterization of CD133+ HPCs from human umbilical cord blood

**Fig. 1:**
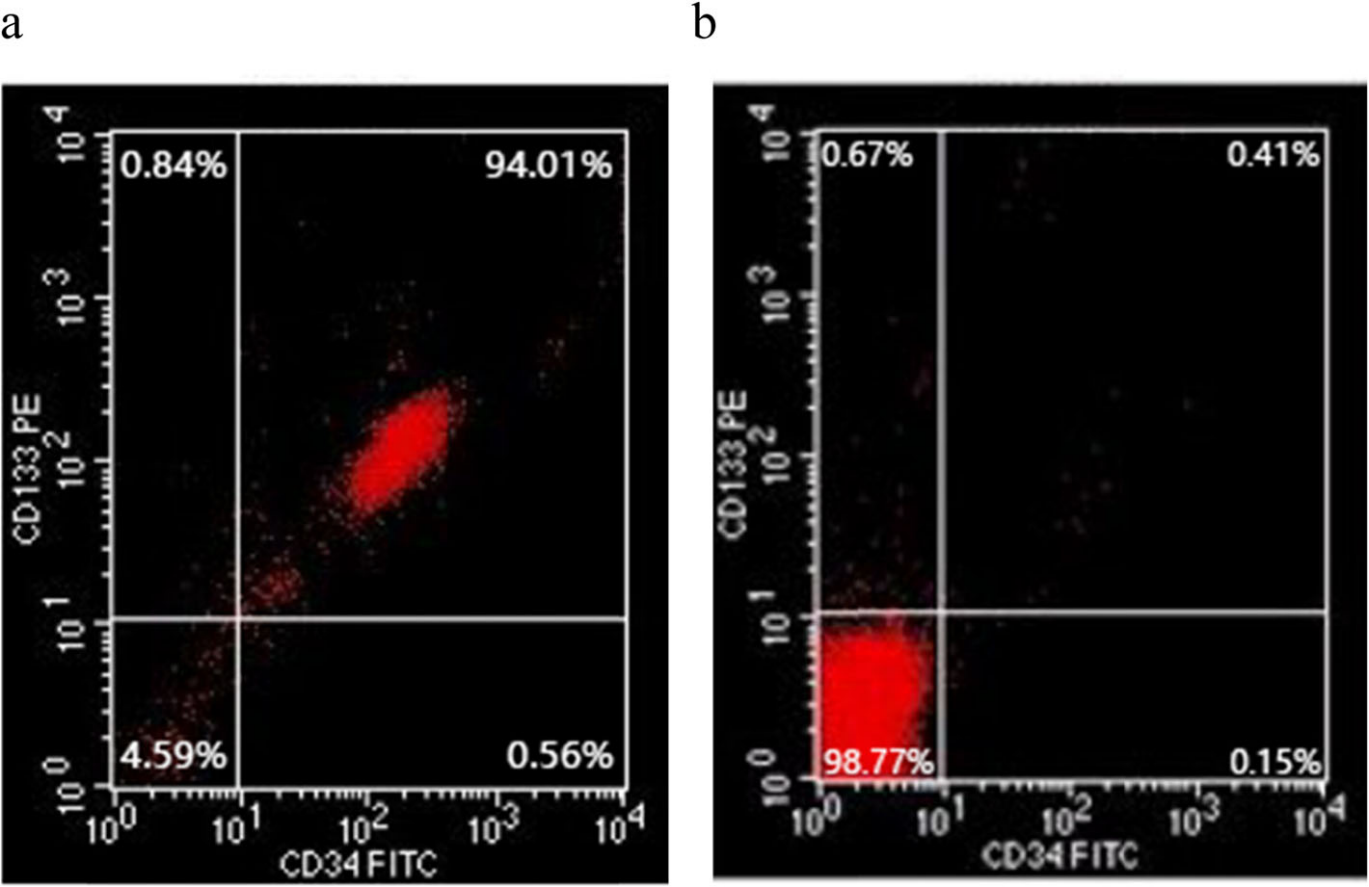
Isolation and identification of human CD133 + CD34+ HPCs. Flow cytometry analysis showing the purity of CD133+ HPCs (**a**); and CD133- HUCBCs (**b**)

To determine the role of CD133+ HPCs in the malignancy of breast cancer cells, we isolated mononuclear cells from five UCB samples and obtained mononuclear cells of (3.07 ± 0.63) × 10^7^/ml. Following magnetic bead-based purification, we collected (2.91 ± 0.6) × 10^5^/ml HPCs, accounting for (0.95 ± 0.011) % of all mononuclear cells. Flow cytometry analysis indicated that the purity of CD133 + CD34+ HPCs was (83 ± 12) % (Fig. 1a and b). Hence, the isolated CD133 + CD34+ HPCs had a high purity.

### CD133+ HPCs promote breast cancer cell proliferation and inhibit their apoptosis in vitro

**Fig. 2:**
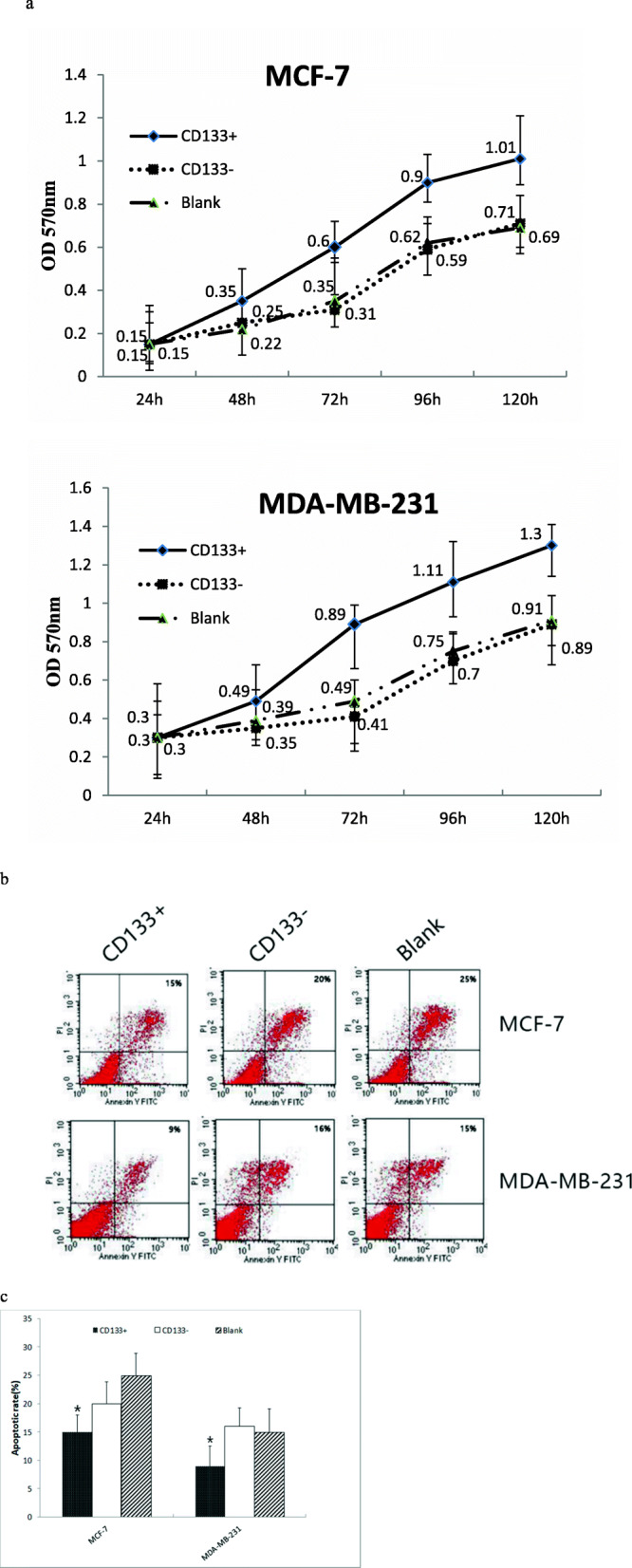
CD133+ HPCs enhances breast cancer cell proliferation and reduces their apoptosis in vitro. Data show representative flow cytometry charts, or present the mean ± SD of each group from three separate experiments. **a** The proliferation of cells; **b** The images of apoptotic cells; **c** Quantitative analysis of apoptotic cells. *P < 0.05, compared with CD133- HUCBCs group

Breast cancer cells display their malignancy by rapid proliferation and potent invasion. Next, we examined the effect of CD133+ HPCs on the proliferation of breast cancer cells in vitro. We cultured MCF-7 or MDA-MB-231 cells alone or together with CD133+ HPCs or CD133- HUCBCs at a cell number ratio of 20:1 for varying time periods, and we quantified breast cancer cell proliferation longitudinally by MTT assay. As shown in Fig. 2a, we observed co-culture with CD133+ HPCs significantly promoted the proliferation of MCF-7 and MDA-MB-231 cells in a time-dependent manner. While there was no significant difference in the growth rate between the cells cultured alone or co-cultured with CD133- HUCBCs in each cell line. Subsequently, we tested whether co-culture with CD133+ HPCs could modulate the spontaneous apoptosis of breast cancer cells by flow cytometry (Fig. 2b and c). We found that the percentages of apoptotic MCF-7 or MDA-MB-231 cells following co-culture with CD133+ HPCs (15% ± 3, 9% ± 3.8 respectively) were significantly lower than that of culture alone or co-culture with CD133- HUCBCs (20% ± 3.5, 16% ± 3.2 respectively; *P* < 0.05 for all). Hence, CD133+ HPCs significantly promoted breast cancer cell proliferation and inhibited their spontaneous apoptosis in vitro.

### CD133+ HPCs enhance the growth of breast cancer in vivo

**Fig. 3:**
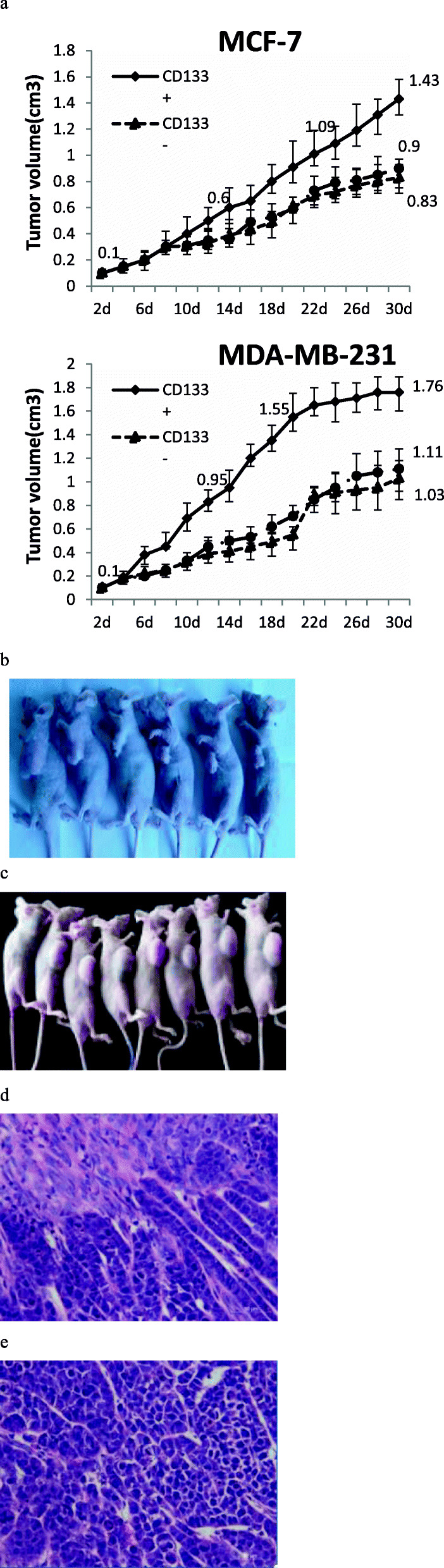
CD133+ HPCs promotes breast cancer growth in vivo. Balb/c nude mice were implanted subcutaneously with MCF-7 or MDA-MB-231 cells alone or together with CD133- HUCBCs or CD133+ HPCs (20:1) and the grown tumor volumes were monitored. **a** Data are present the mean ± SD of each group (*n* = 8); **b** Tumor formation of MCF-7 cells in vivo; **c** Tumor formation of MDA-MB-231 cells in vivo; **d** Representative histology (H&E) for the MCF-7 tumor tissue (× 400); **e** Representative histology (H&E) for the MDA-MB-231 tumor tissue (× 400)

We further evaluated the effect of CD133+ HPCs on the growth of breast cancer in vivo. Following inoculation of MCF-7 or MDA-MB-231 cells alone, or together with CD133+ HPCs or CD133- HUCBCs at a ratio of 20:1, we monitored the dynamic growth of implanted tumors by measuring their volumes (Fig. 3a). At day 30, co-administration of breast cancer cells with CD133+ HPCs (MCF-7 1.43cm^3^ ± 0.15, MDA-MB-231 1.76 cm^3^ ± 0.13) significantly increased tumor volumes when compared to that of the mice with breast cancer alone and the cells mixed with CD133- HUCBCs (MCF-7 0.83cm^3^ ± 0.12, MDA-MB-231 1.03 cm^3^ ± 0.16; *P* < 0.05 for all). In contrast, there were no significant difference in tumor size between the mice with breast cancer alone and the cells mixed with CD133- HUCBCs. Thus, CD133+ HPCs enhanced the growth of implanted breast tumors in vivo. Histologic examination of two types of tumors are shown in Fig. 3b and c. The parenchymal cells in the cancer tissue were much more than those in the stroma, and the cancer cells were arranged in a strip like manner, in which scattered tumor cells were observed. The tumor cells were irregular with a large volume and large nucleus. The nucleoli were clear and pleomorphic. The pathological diagnosis was invasive ductal carcinoma (Fig. 3d and e).

### CD133+ HPCs promote breast cancer cell invasion in vitro

**Fig. 4:**
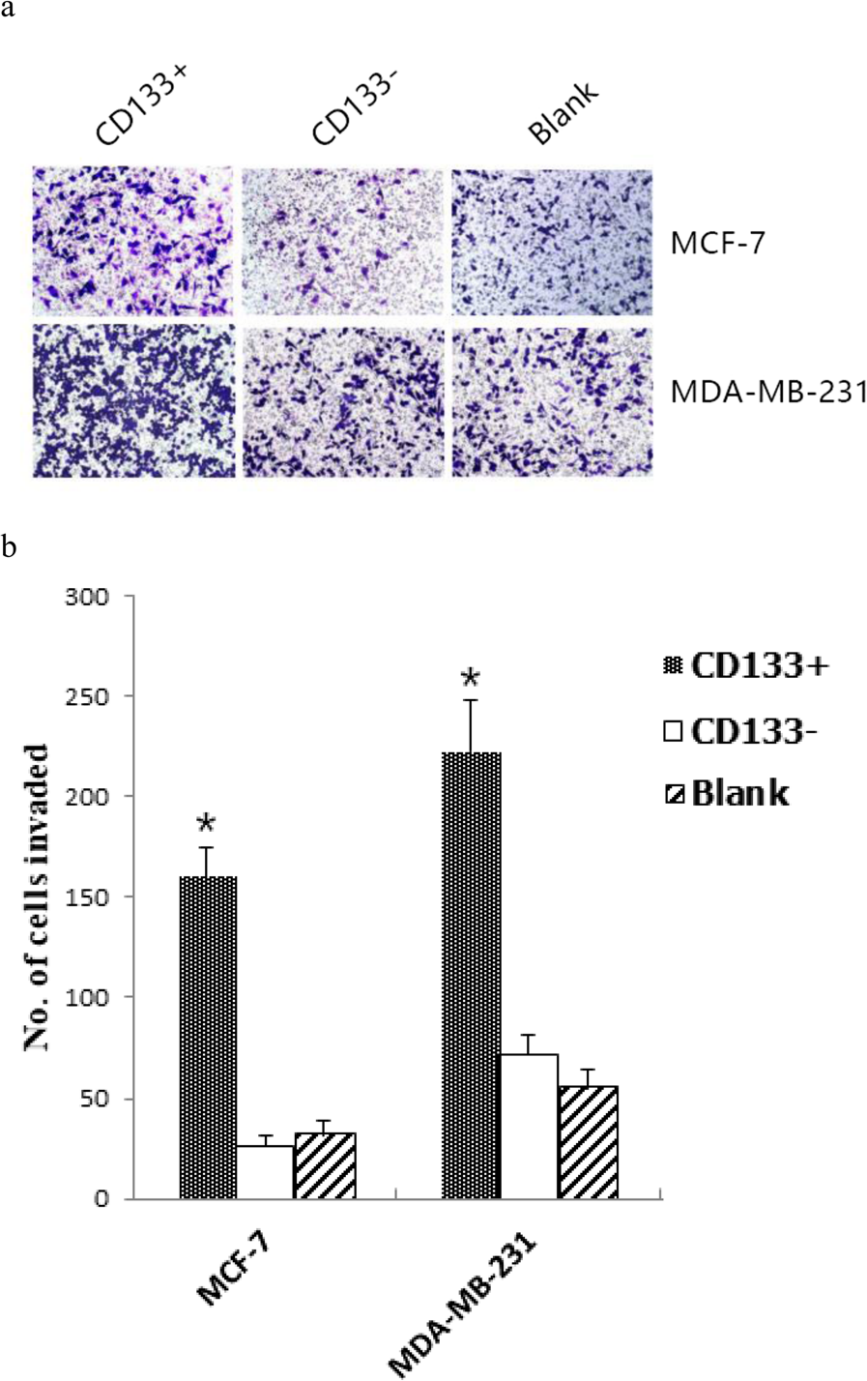
CD133+ HPCs enhances breast cancer cell invasion in vitro. The Transwell assay was conducted and the invasive breast cancer cells were stained, photoimaged and counted. **a** A representative image of the invasive cells (magnification × 400). **b** Quantitative analysis of invasive cells. *P < 0.05, compared with CD133- HUCBCs group

Breast cancer cell invasion is one of the malignant characteristics. To understand the role of CD133+ HPCs, we tested whether CD133+ HPCs could modulate breast cancer cell invasion by Transwell invasion assays. Following culture of each type of breast cancer cells alone, with CD133+ HPCs or CD133- HUCBCs separately in Transwell chambers for 24 h, we found that the numbers of invaded cells that had been cultured with CD133+ HPCs (MCF-7160 cells±15, MDA-MB-231222 cells±5) were significantly greater than that of the cells cultured alone or with CD133- HUCBCs (MCF-7 26 cells±11, MDA-MB-231 72 cells±9; *P* < 0.05 for all, Fig. 4a and b) in two different lines. We did not observe any significant difference in the numbers of invaded cells between the cell cultured alone and those with CD133- HUCBCs in our experimental system. Therefore, CD133+ HPCs enhanced breast cancer cell invasion in vitro.

### CD133+ HPCs enhances the process of EMT in breast cancer cells

**Fig. 5:**
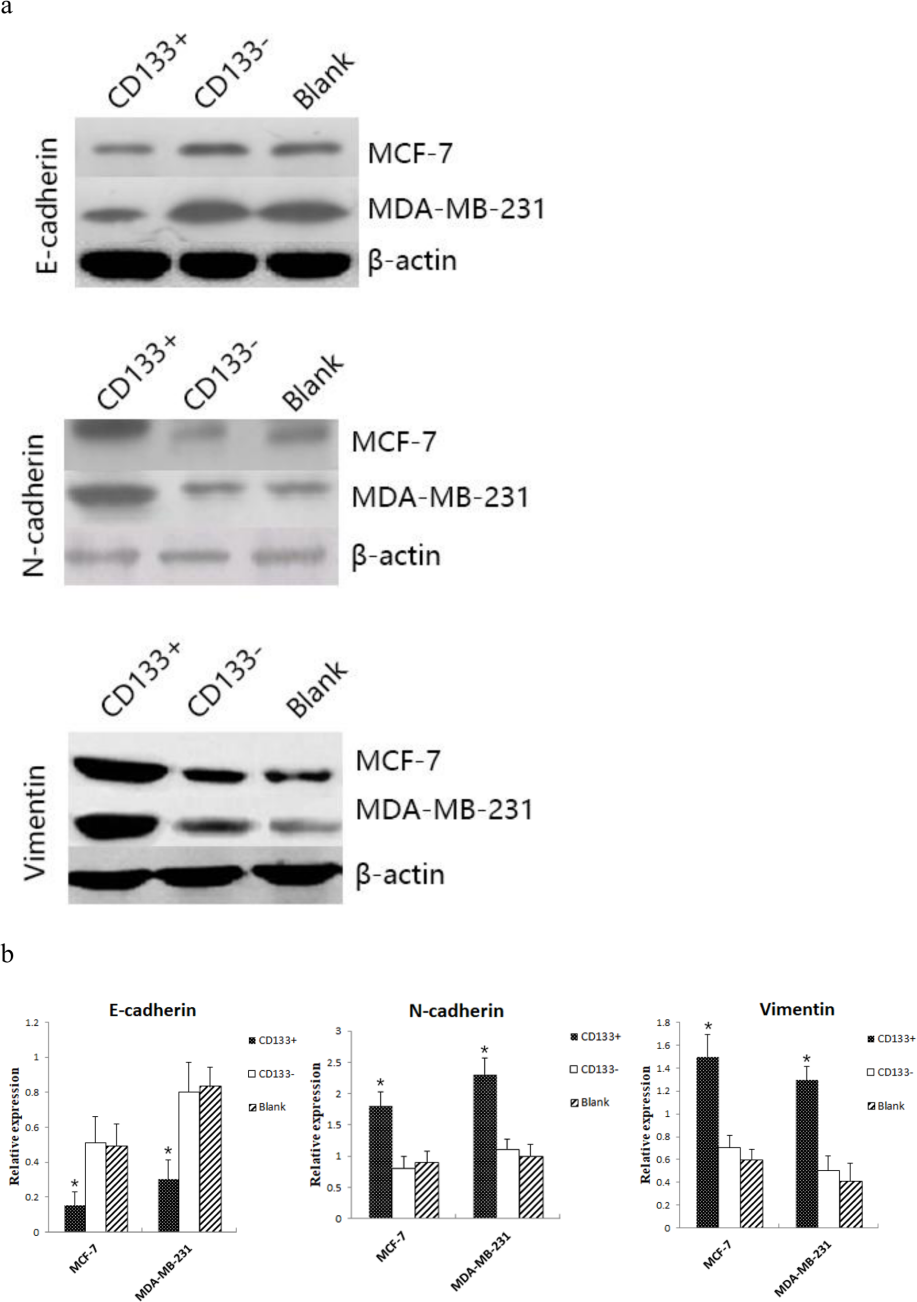
CD133+ HPCs promotes the EMT process in breast cancer cells. Breast cancer cells were culture alone or co-culture with CD133- HUCBCs or CD133+ HPCs in Transwell plates, the relative levels of E-cadherin, N-cadherin and Vimentin to the control β-actin expression in breast cancer cells were quantified by Western blot. Data are representative images or present as the mean ± SD of each group from three separate experiments. **a** Western blot analysis. **b** Quantitative analysis of each protein expression. *P < 0.05, compared with CD133- HUCBCs group

The EMT process is associated with invasion and metastasis of cancers. Therefore, we further explored whether CD133+ HPCs could affect the EMT process in breast cancer cells to promote invasion by quantifying the relative levels of E-cadherin, N-cadherin and Vimentin expression using Western blot assays. The results showed that the expression of E-cadherin in MCF-7 and MDA-MB-231 breast cancer cells co-cultured with CD133+ HPCs (0.15 ± 0.08, 0.3 ± 0.11 respectively) was significantly lower than that co-cultured with CD133- HPCs (0.51 ± 0.15, 0.8 ± 0.17 respectively) and in breast cancer cells alone (0.49 ± 0.13, 0.83 ± 0.11 respectively, *P* < 0.05). The expression of N-cadherin in MCF-7 and MDA-MB-231 breast cancer cells co-cultured with CD133+ HPCs (1.8 ± 0.23, 2.3 ± 0.27 respectively) was significantly higher than that of co-cultured with CD133- HPCs (0.8 ± 0.19, 1.1 ± 0.17 respectively) and single breast cancer cells (0.9 ± 0.18, 1 ± 0.19 respectively; *P* < 0.05). The expression of Vimentin in MCF-7 and MDA-MB-231 breast cancer cells co-cultured with CD133+ HPCs (1.5 ± 0.19,1.3 ± 0.11 respectively) was significantly higher than that of co-cultured with CD133- HPCs (0.7 ± 0.11, 0.5 ± 0.13 respectively) and single breast cancer cells (0.6 ± 0.09, 0.41 ± 0.16 respectively; *P* < 0.05). We found that there were similar levels of E-cadherin, N-cadherin and Vimentin expression in the cells cultured alone or together with CD133- HUCBCs (Fig. 5a and b). In comparison with the control, significantly down-regulated E-cadherin expression, but up-regulated N-cadherin and Vimentin expression were observed in the cells co-cultured with CD133+ HPCs (*P* < 0.05 for all). Such data indicated that CD133+ HPCs promoted the EMT process in breast cancer cells.

## Discussion

VEGFR1+ HPCs can promote tumor metastasis in rodent models of cancers [5]. In this study, we purified human CD133 + CD34+ HPCs from UCB.

We found that co-culture of CD133+ HPCs, but not CD133- HUCBCs, remarkably strengthened breast cancer cell proliferation and invasion, accompanied by attenuating spontaneous apoptosis in vitro and enhanced breast tumor growth in vivo. The lack of modulatory effect of CD133- HUCBCs suggests that CD133 expression is an important marker for human HPCs. The increased proliferation and invasion as well as tumor growth in vivo by CD133+ HPCs indicated that CD133+ HPCs enhanced the malignancy of different types of breast cancers. Given that CD133+ HPCs enhanced breast cancer cell invasion in Transwell plates, the promoting activity of CD133+ HPCs may be mediated by secreting soluble oncogenic factors. Future studies are necessary to investigate which soluble molecules CD133+ HPCs secrete and how they enhance the malignancy of breast cancers. At the same time, the molecular and cellular changes might be induced in breast cancer cells in the presence of CD133+ HPCs have not been uncovered,the follow-up experimental plan of our research group is to identify possible pathways of action and study the changes of pathway which effected by the antibodies that act on CD133 + targets.

It is well known that the EMT process is associated with cancer invasion and metastasis [27]. In this study, we found that co-culture of CD133+ HPCs, but not CD133- HUCBCs significantly decreased E-cadherin, but increased N-cadherin and Vimentin expression in breast cancer cells, indicating that CD133+ HPCs promoted the EMT process in breast cancer cells. The increased EMT process by CD133+ HPCs may also mechanistically explain how CD133+ HPCs enhance the invasion of breast cancer cells.

## Conclusions

In summary, we determined that CD133+ HPCs remarkably strengthened the malignancy of breast cancer cells by enhancing their proliferation and invasion, accompanied by attenuating spontaneous apoptosis in vitro and enhancing breast tumor growth in vivo. Furthermore, CD133+ HPCs increased the EMT process in breast cancer cells. Given that CD133+ HPCs can circulate in peripheral blood, such type of cells may contribute to the pathological process of breast cancer. Therefore, CD133+ HPCs may be new targets for therapies against breast cancer metastasis and invasion.

## Supplementary Information

**Additional file 1 **: **Figure 1**. a. The relative levels of E-cadherin to the control β-actin expression in breast cancer cells were quantified by Western blot. b. The relative levels of N-cadherin to the control β-actin expression in breast cancer cells were quantified by Western blot. c. The relative levels of Vimentin to the control β-actin expression in breast cancer cells were quantified by Western blot.

## Data Availability

The data to support the findings of this study are available upon reasonable request from the corresponding author, but restrictions apply to the availability of these data, which were used under license for the current study, and so are not publicly available.

## Abbreviations

HPCs: Hematopoietic progenitor cells
UCB: Umbilical cord blood
EMT: Epithelial mesenchymal transition
HUCBCs: Human umbilical cord blood cells
MTT: 3-(4,5-dimethylthiazol-2-yl)-2,5-diphenyltetrazolium bromide
