# Supplementary material for: Human CD133-positive hematopoietic progenitor cells enhance the malignancy of breast cancer cells
Source: BMC Cancer. 2020 Nov 26;20:1158. doi: 10.1186/s12885-020-07633-3 (PMC7690192; doi:10.1186/s12885-020-07633-3)
Supplement: Supplementary file 1 — Additional file 1 : Figure 1. a. The relative levels of E-cadherin to the control β-actin expression in breast cancer cells were quantified by Western blot. b. The relative levels of N-cadherin to the control β-actin expression in breast cancer cells were quantified by Western blot. c. The relative levels of Vimentin to the control β-actin expression in breast cancer cells were quantified by Western blot. [file 12885_2020_7633_MOESM1_ESM.docx]

Fig.1

a.The relative levels of E-cadherin to the control β-actin expression in breast cancer cells were quantified by Western blot.


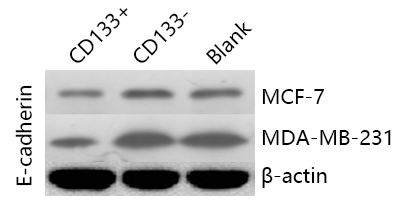


**Original figure:**

**CD133+**

**Blank**

**CD133-**


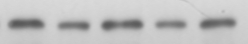


**MCF-7**

**CD133+**

**Blank**

**CD133-**

**MDA-MB-231**


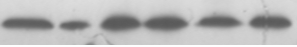


**Blank**

**CD133-**

**CD133+**


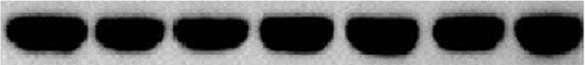


**β-actin**

b.The relative levels of N-cadherin to the control β-actin expression in breast cancer cells were quantified by Western blot.


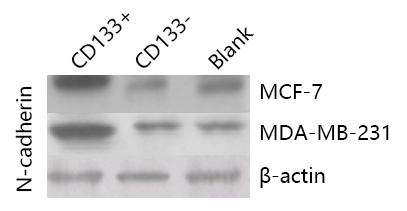


**Original figure:**

**CD133-**

**CD133+**

**Blank**


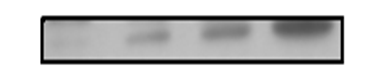


**MCF-7**

**Blank**

**CD133-**

**CD133+**


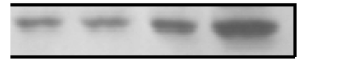


**MDA-MB-231**

**Blank**

**CD133-**

**CD133+**


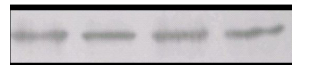


**β-actin**

c.The relative levels of Vimentin to the control β-actin expression in breast cancer cells were quantified by Western blot.


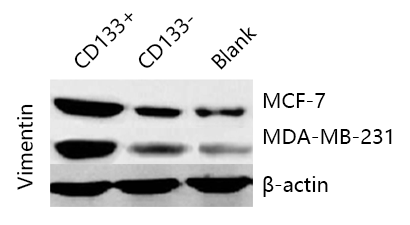


**Original figure:**

**Blank**

**CD133-**

**CD133+**


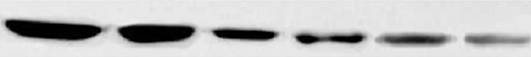


**MCF-7**

**CD133-**

**CD133+**

**Blank**


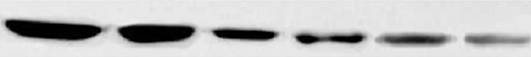


**MDA-MB-231**

**Blank**

**CD133-**

**CD133+**


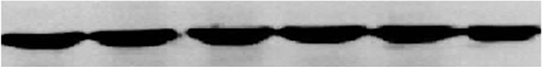


**β-actin**
